# Generalized (*σ* , *τ*) higher derivations in prime rings

**DOI:** 10.1186/2193-1801-1-31

**Published:** 2012-10-06

**Authors:** Mohammad Ashraf, Almas Khan

**Affiliations:** Department of Mathematics, Aligarh Muslim University, Aligarh, 202002 India

**Keywords:** Derivation, Higher derivation, Jordan - higher derivation, Lie ideal

## Abstract

Let *R* be a ring and *U* be a Lie ideal of *R*. Suppose that *σ*, *τ* are endomorphisms of *R*. A family *D* = {*d*_*n*_}_*n* ∈ **N**_of additive mappings *d*_*n*_:*R* → *R* is said to be a (*σ*,*τ*)- higher derivation of *U* into *R* if *d*_0_ = *I*_*R*_, the identity map on *R* and  holds for all *a*, *b* ∈ *U* and for each *n* ∈ **N**. A family *F* = {*f*_*n*_}_*n* ∈ **N**_of additive mappings *f*_*n*_:*R* → *R* is said to be a generalized (*σ*,*τ*)- higher derivation (resp. generalized Jordan (*σ*,*τ*)-higher derivation) of *U* into *R* if there exists a (*σ*,*τ*)- higher derivation *D* = {*d*_*n*_}_*n* ∈ **N**_of *U* into *R* such that, *f*_0_ = *I*_*R*_ and  (resp.  holds for all *a*, *b* ∈ *U* and for each *n* ∈ **N**. It can be easily observed that every generalized (*σ*,*τ*)-higher derivation of *U* into *R* is a generalized Jordan (*σ*,*τ*)-higher derivation of *U* into *R* but not conversely. In the present paper we shall obtain the conditions under which every generalized Jordan (*σ*,*τ*)- higher derivation of *U* into *R* is a generalized (*σ*,*τ*)-higher derivation of *U* into *R*.

## Introduction

Throughout (*σ*,*τ*) the present paper *R* will denote an associative ring with center *Z*(*R*). For any *x*,*y* ∈ *R* denote the commutator *xy* − *yx* by [*x*,*y*]. Recall that a ring *R* is prime if *aRb* = {0} implies that *a* = 0 or *b* = 0. An additive subgroup *U* of *R* is said to be a Lie ideal of *R* if [*U*,*R*] ⊆ *U*. A Lie ideal *U* of *R* is said to be a square closed Lie ideal of *R* if *u*^2^∈*U* for all *u*∈*U*. An additive mapping *d*:*R*→*R* is said to be a derivation (resp. Jordan derivation) of *R* if *d*(*xy*) = *d*(*x*)*y* + *xd*(*y*)(resp. *d*(*x*^2^) = *d*(*x*)*x* + *xd*(*x*)) holds for all *x*,*y* ∈ *R*. Now let *D* = {*d*_*n*_}_*n* ∈ **N**_ be a family of additive mappings *d*_*n*_:*R*. Following Hasse and Schimdt (→ *R*^1937^), *D* is said to be a higher derivation (resp. Jordan higher derivation) on *R* if *d*_0_ = *I*_*R*_(the identity map on *R*) and  (resp.  holds for all *a*, *b* ∈ *R* and for each *n* ∈ **N**. Several interesting results on higher derivation can be seen in Haetinger (2000). Let *σ*,*τ* be endomorphisms of *R*. An additive mapping *d*:*R*→*R* is said to be a (*σ*,*τ*)-derivation (resp. Jordan (*σ*,*τ*)-derivation) of *R* if *d*(*xy*) = *σ*(*x*)*d*(*y*) + *d*(*x*)*τ*(*y*) (resp. *d*(*x*^2^) = *σ*(*x*)*d*(*x*) + *d*(*x*)*τ*(*x*)) holds for all *x*,*y* ∈ *R*. For a fixed *a* ∈ *R*, the map *d*_*a*_:*R*→*R*given by *d*_*a*_(*x*) = *aτ* (*x*) − *σ*(*x*)*a* for all *x* ∈ *R* is a (*σ*,*τ*)- derivation which is said to be a (*σ*,*τ*)-inner derivation determined by *a*.

Inspired by the notion of (*σ*,*τ*)- derivation the authors together with Haetinger (2010) introduced the concept of a (*σ*,*τ*)- higher derivation as follows: A family *D* = {*d*_*n*_}_*n* ∈ **N**_ of additive mappings *d*_*n*_:*R* → *R* is said to be a (*σ*,*τ*)- higher derivation of *R* if *d*_0_ = *I*_*R*_ and  holds for all *a*, *b* ∈ *R* and for each *n* ∈ **N** (If *U* is a Lie ideal of *R*, then *D* is said to be a (*σ*,*τ*)- higher derivation of *U* into *R* if the corresponding conditions are satisfied for all *a*, *b* ∈ *U*).

Following Bres̆ar (1991), an additive mapping *F*:*R*→*R*is said to be a generalized derivation if there exists a derivation *d*:*R*→*R* such that *F*(*xy*) = *F*(*x*)*y* + *xd*(*y*) holds for all *x*, *y* ∈ *R*. Motivated by the definition of generalized derivation the notion of generalized higher derivation was introduced by Cortes and Haetinger (2005). A family *F* = {*f*_*n*_}_*n* ∈ **N**_of additive maps *f*_*n*_:*R*→*R*is said to be a generalized higher derivation of *R* if there exists a higher derivation *D* = {*d*_*n*_}_*n* ∈ **N**_ of *R* such that *f*_0_ = *I*_*R*_ and  for all *a*, *b* ∈ *R* and for each *n* ∈ **N**.

An additive mapping *F*:*R*→*R* is said to be a generalized (*σ*,*τ*)-inner derivation if *F*(*x*) = *σ*(*x*)*b* + *aτ*(*x*) holds for some fixed *a*, *b* ∈ *R* and for all *x* ∈ *R*. If *F* is a generalized (*σ*,*τ*)-inner derivation, a simple computation yields that *F*(*xy*) = *σ*(*x*)*d*_−*b*_(*y*) + *F*(*x*)*τ*(*y*), where *d*_−*b*_ is a (*σ*,*τ*)-inner derivation. With this point of view an additive mapping *F*:*R*→*R* is said to be a generalized (*σ*,*τ*)-derivation on *R* if there exists a (*σ*,*τ*)-derivation *d*:*R*→*R* such that *F*(*xy*) = *σ*(*x*)*d*(*y*) + *F*(*x*)*τ*(*y*) holds for all *x*,*y* ∈ *R*. For such an example let *S* be any ring and . Define an additive map *F*:*R*→*R* such that  and endomorphisms *σ*,*τ*:*R*→*R* such that  and . Then it can be easily seen that *F* is a generalized (*σ*,*τ*)-derivation on *R* with associated (*σ*,*τ*)- derivation *d*:*R*→*R* such that .

In view of the above definition we introduce the analogue of (*σ*,*τ*)-higher derivation in a more general setting.

Let *σ*,*τ* be endomorphims of *R*. A family *F* = {*f*_*n*_}_*n* ∈ **N**_ of additive maps *f*_*n*_:*R*→*R* is said to be generalized (*σ*,*τ*)-higher derivation (resp. generalized Jordan (*σ*,*τ*)-higher derivation) of *R* if there exists a (*σ*,*τ*)-higher derivation *D* = {*d*_*n*_}_*n* ∈ **N**_ of *R* such that *f*_0_ = *I*_*R*_ and  (resp.  holds for all *a*, *b* ∈ *R* and for each *n* ∈ **N**.

Let *U* be a Lie ideal of *R*. Then *F* is said to be a generalized (*σ*,*τ*)-higher derivation (resp. generalized Jordan (*σ*,*τ*)-higher derivation) of *U* into *R* if the above corresponding conditions are satisfied for all *a*, *b* ∈ *U*.

**Example 1.1.** Let *R* be an algebra over the field of rationals and *σ*,*τ* be the endomorphisms of *R*. Define , for all *n* ∈ **N**, where *f* is a generalized (*σ*,*τ*)- derivation on *R* with associated (*σ*,*τ*)-derivation *δ*such that *fσ* = *σf* and *δτ* = *τδ*(the above example of generalized (*σ*,*τ*)-derivation ensures the existence of such *f*). Consider the sequence {*F*_*n*_}_*n* ∈ **N**_, this defines a generalized (*σ*,*τ*)- higher derivation with associated (*σ*,*τ*)-higher derivation .

If we choose the underlying *f* to be a generalized Jordan (*σ*,*τ*)−derivation on *R* which is not a generalized (*σ*,*τ*)−derivation on *R* then one can easily construct an example of a generalized Jordan (*σ*,*τ*)− higher derivation on *R* which is not a generalized (*σ*,*τ*)− higher derivation on *R*.

It is easy to see that every derivation on *R* is a Jordan derivation but the converse need not be true in general. A well-known result due to Herstein (2002) states that every Jordan derivation on a prime ring of characteristic different from two is a derivation. This result was further generalized by many authors in various directions (see Ashraf et al. 2001; Bres̆ar and Vukman 1991where further references can be found). Motivated by these results Ferrero and Haetinger (2002) generalized Herstein’s theorem for higher derivations and proved that every Jordan higher derivation on a prime ring of characteristic different from two is a higher derivation. The authors together with Haetinger (Ashraf et al. 2010) further generalized the above result in the setting of (*σ*,*τ*)-higher derivation of *R*. The main objective of the present paper is to find the conditions on *R* under which every generalized Jordan (*σ*,*τ*)-higher derivation of *R* is a generalized (*σ*,*τ*)-higher derivation of *R*. In fact our results generalize, extend and compliment several results obtained earlier in this direction.

## Main results

Recently, Haetinger (2002) proved that if *R* is a prime ring of characteristic different from 2 and *U* a square closed Lie ideal such that *U*⫅̸*Z*(*R*). Then every Jordan higher derivation of *U* into *R* is a higher derivation of *U* into *R*. The following theorem shows that the above result still holds for arbitrary square closed Lie ideal of *R* that is, *U* may be central.

**Theorem 2.1.** Let *R* be a prime ring such that *char*(*R*) ≠ 2 and *U* be a square closed Lie ideal of *R*. Suppose that *σ*,*τ* are endomorphisms of *R* such that *στ* = *τσ* and *τ* is one-one & onto. Then every generalized Jordan (*σ*,*τ*)-higher derivation of *U* into *R* is a generalized (*σ*,*τ*)-higher derivation of *U* into *R*.

In order to develop the proof of the theorem, we begin with the following known lemma:

**Lemma 2.1.** ((Ferrero and Haetinger 2002), Lemma 2.3) Assume that *R* is a 2-torsion free prime ring and *U* a square closed Lie ideal of *R* such that *U*⫅̸*Z*(*R*). Let *G*_1_,*G*_2_,⋯,*G*_*n*_be additive groups, *S*:*G*_1_×*G*_2_×⋯×*G*_*n*_→*R* and *T*:*G*_1_×*G*_2_×⋯×*G*_*n*_→ *R* be the mappings which are additive in each argument. If *S*(*a*_1_,*a*_2_,⋯,*a*_*n*_)*xT*(*a*_1_,*a*_2_,⋯,*a*_*n*_) = 0 for every *x* ∈ *U*, *a*_*i*_∈*G*_*i*_, *i* = 1,2,⋯,*n* then *S*(*a*_1_,*a*_2_,⋯,*a*_*n*_) = 0 for every *a*_*i*_∈*G*_*i*_, *i* = 1,2,⋯,*n*or *T*(*b*_1_,*b*_2_,⋯,*b*_*n*_) = 0 for every *b*_*i*_∈*G*_*i*_, *i* = 1,2,⋯,*n*.

**Lemma 2.2.** Let *R* be a ring and *σ*, *τ* be endomorphisms of *R* such that *στ* = *τσ* and *F* = {*f*_*n*_}_*n* ∈ **N**_be a generalized Jordan (*σ*,*τ*)-higher derivation of *U* into *R* with associated (*σ*,*τ*)-higher derivation *D* = {*d*_*n*_}_*n* ∈ **N**_ of *U* into *R*. Then for all *u*, *v*, *w* ∈ *U* and each fixed *n* ∈ **N** we have for all *u*, *v*, *w* ∈ *U*.

(i) 

If *R* is a 2-torsion free ring then,

(ii) 

(iii) 

*Proof.* (i) For *u*, *v* ∈ *U*, *n* ∈ **N** we have, .By linearizing the above relation on *u* we obtain 

 for all *u*, *v* ∈ *U*. Again; 

 for all *u*, *v* ∈ *U*.Comparing the above expressions and reordering the indices we obtain the required result.

(ii) Since *uv* + *vu* = (*u* + *v*)^2^−*u*^2^−*v*^2^∈*U*, using (*i*) and replacing *v* by *uv* + *vu* we find that, 2.1

On the other hand, 2.2

Comparing the equations (2.1) and (2.2) and reordering the indices and using the fact that *R* is 2-torsion free we get the required result.

(iii) Linearizing the above result, we have 2.3

Again, 2.4

Comparing (2.3) & (2.4) and using the fact that *R* is 2-torsion free we get the required result.

For every fixed *n* ∈ **N** and for each *u*,*v*∈*U* we denote by *Φ*_*n*_(*u*,*v*) the element of *R* such that . It is straight forward to see that if *Φ*_*n*_(*u*,*v*) = 0, then *F* = {*f*_*n*_}_*n* ∈ **N**_ is a generalized (*σ*,*τ*)-higher derivation of *U* into *R*. Trivially, by using Lemma 2.2(*i*) we get *Φ*_*n*_(*u*,*v*) = −*Φ*_*n*_(*v*,*u*), for all *u*,*v*∈*U*.

It can also be seen that the function *Φ*_*n*_is additive in both the arguments.

**Lemma 2.3.** Let *R* be a 2-torsion free ring and *σ*,*τ*be endomorphisms of *R* such that *στ* = *τσ*. Let *F* = {*f*_*n*_}_*n* ∈ **N**_be a generalized Jordan (*σ*,*τ*)-higher derivation of *U* into *R* with associated (*σ*,*τ*)-higher derivation *D* = {*d*_*n*_}_*n* ∈ **N**_of *U* into *R*. If *Φ*_*m*_(*u*,*v*) = 0, for each *m*<*n* and for all *u*,*v*∈*U*, then 

(i) *Φ*_*n*_(*u*,*v*)*τ*^*n*^[*u*,*v*] = 0, for all *u*,*v*∈*U*

(ii) *Φ*_*n*_(*u*,*v*)*τ*^*n*^(*w*)*τ*^*n*^[*u*,*v*] = 0, for all *u*,*v*,*w*∈*U*.

*Proof.*

(i) Since for any *u*,*v*∈*U*, *uv* + *vu*∈*U* and *uv*−*vu*∈*U*, we find that 2*uv*∈*U*. Suppose *β* = 4(*uv*(*uv*) + (*uv*)*vu*)∈*U*. Using Lemma 2.2(*iii*) we have, 

Using the fact that *Φ*_*m*_(*u*,*v*) = 0 for all *m*<*n* we have, 2.5

On the other hand, 2.6

Comparing (2.5) with (2.6) for *f*_*n*_(*β*)we get *Φ*_*n*_(*u*,*v*) × [*τ*^*n*^(*u*),*τ*^*n*^(*v*)] = 0 for all *u*, *v* ∈ *U*.

(ii) Let *χ* = 4(*uvwvu* + *vuwuv*) for *u*, *v*, *w* ∈ *U*. Then by Lemma 2.2(*ii*) we obtain 2.7

Again consider, 

Applying Lemma 2.2(*iii*), we have 2.8

Equating (2.7) & (2.8) and using the fact that *R* is 2 torsion free we find that 2.9

Initially calculating the first term we have 

Using the hypothesis that *Φ*_*m*_(*u*,*v*) = 0 for all *m* < *n*. 2.10

Similarly the second term reduces to 2.11

Now, subtracting the equation (2.11) from (2.10) and using the hypothesis that *στ* = *τσ* we obtain 

Similarly, the difference of the last two terms of equation (2.9) yields 

Thus, (2.9) becomes *Φ*_*n*_(*u*,*v*)*τ*^*n*^(*w*)*τ*^*n*^[*u*,*v*] = 0 for all *u*, *v* ∈ *U.*

We are now well equipped to prove our main theorem.

*Proof of Theorem 2.1.* Suppose that *U* is commutative. If *U* is commutative then *U* is also central (see the proof of Lemma 1.3 of (Herstein 1969)). We’ll proceed by induction on *n*. For *n* = 1, every generalized Jordan (*σ*,*τ*)-higher derivation reduces to generalized Jordan (*σ*,*τ*)-derivation and hence using Lemma 2.2(*iii*) of (Ashraf et al. 2001), we have 2.12

As *U* is commutative, in view of Lemma 2.2(*i*) of (Ashraf et al. 2001) we have 

Since, *d*(*uv*) = *d*(*vu*) = *σ*(*v*)*d*(*u*) + *d*(*v*)*τ*(*u*), the above equation can be rewritten as 2.13

Comparing the equations (2.12) and (2.13) we obtain 

As *w* is central and since *τ* is one-one and onto hence *τ*(*w*) is central but the center of a prime ring is free from zero divisors, the above equation implies that *Φ*_1_(*u*,*v*) = 0 for all *u*, *v* ∈ *U*. Let *Φ*_*m*_(*u*,*v*) = 0 for all *u*, *v* ∈ *U* and each *m* < *n* then from Lemma 2.2(*iii*), we have 2.14

for all *u*, *v*, *w* ∈ *U*.

Again by using Lemma 2.2(*i*) and commutativity of *U*, we get 

Since *U* is commutative we find that *d*_*j*_(*τ*^*n*−*j*^(*uv*)) = *d*_*j*_(*τ*^*n*−*j*^(*vu*)) and as *d*_*j*_ is a (*σ*,*τ*)-higher derivation the above equation can be written as 2.15

Comparing equations (2.15) and (2.14) we find that 

Since *Φ*_*m*_(*u*,*v*) = 0 for all *u*, *v* ∈ *U*, *m* < *n*, the above equation reduces to 2.16

Since *w* is central and as *τ* is one-one and onto, *τ*^*n*^(*w*) is also central. But the center of a prime ring is free from the zero divisors, equation (2.16) implies that *Φ*_*n*_(*u*,*v*) = 0 for all *u*, *v* ∈ *U* and each *n* ∈ **N**.

Now consider the possibility that *U* is non-commutative hence *U*⫅̸*Z*(*R*). Using Lemma 2.3(*ii*) we have *Φ*_*n*_(*u*,*v*)*τ*^*n*^(*w*)*τ*^*n*^[*u*,*v*] = 0 for all *u*,*v*,*w*∈*U*. Since *τ* is one-one and onto, the relation yields that *τ*^−*n*^(*Φ*_*n*_(*u*,*v*))*w*[*u*,*v*] = 0 for all *u*,*v*,*w*∈*U*. Hence by Lemma 2.1, *τ*^−*n*^(*Φ*_*n*_(*u*,*v*)) = 0 for all *u*,*v*∈*U* or [*u*,*v*] = 0 for all *u*,*v*∈*U*. But since *U* is non-commutative, we find that *Φ*_*n*_(*u*,*v*) = 0, for all *u*,*v*∈*U* and each *n* ∈ **N**.This completes the proof of our theorem.

As a consequence of the above result we find the following corollaries. Corollary 2.1 settle the conjecture given in (Ashraf et al. 2004) for a square closed Lie ideal while Corollary 2.2 improves the main Theorems of (Ashraf et al. (2010), Bres̆ar and Vukman (1988); Haetinger (2002)). □

**Corollary 2.1.** Let *R* be 2 torsion free prime ring and *U* a square closed Lie ideal of *R*. Suppose that *θ*,*ϕ* are endomorphisms of *R* such that *ϕ* is one-one, onto. If *F*:*R*→*R* is a generalized Jordan (*θ*,*ϕ*) derivation on *U* then *F* is a generalized (*θ*,*ϕ*) derivation on *U*.

**Corollary 2.2.** Let *R* be a prime ring such that *char*(*R*) ≠ 2 and *U* a square closed Lie ideal of *R*. Then every Jordan higher derivation of *U* into *R* is a higher derivation of *U* into *R*.

In the above theorem if the underlying ring is arbitrary, then we can prove the following:

**Theorem 2.2.** Let *R* be a 2- torsion free ring and *U* be a square closed Lie ideal of *R*. Suppose that *σ*,*τ* are endomorphisms of *R* such that *στ* = *τσ* and *τ* is one-one & onto. If *U* has a commutator which is not a right zero divisor, then every generalized Jordan (*σ*,*τ*)-higher derivation of *U* into *R* is a generalized (*σ*,*τ*)-higher derivation of *U* into *R*.

*Proof.* Let *x*,*y*∈*U* be the fixed elements such that *c*[*x*,*y*]=0⇒*c* = 0 for every *c*∈*R*. We’ll prove the result by induction on *n*.

We know that for *n* = 0, *Φ*_0_(*u*,*v*) = 0. Hence proceeding by induction we can assume that *Φ*_*m*_(*u*,*v*) = 0 for all *m*<*n*. Using Lemma 2.3(*i*) we have 2.17

The above equation implies that *τ*^−*n*^(*Φ*_*n*_(*u*,*v*))[*u*,*v*] = 0 for all *u*, *v* ∈ *U*. Hence in particular, *τ*^−*n*^(*Φ*_*n*_(*x*,*y*))[*x*,*y*] = 0. This implies that, 2.18

Replacing *u* by *u* + *x* in (2.17) we get 2.19

Again replacing *v* by *y* in (2.19) and using (2.18) we get *Φ*_*n*_(*u*,*y*)[*τ*^*n*^(*x*),*τ*^*n*^(*y*)] = 0, for every *u* ∈ *U*,i.e., *τ*^−*n*^(*Φ*_*n*_(*u*,*y*))[*x*,*y*] = 0. This yields that 2.20

Replace *v* by *v* + *y* in (2.19), to get 2.21

Replacing *u* by *x* in (2.21) and using (2.18) we obtain *Φ*_*n*_(*x*,*v*)[*τ*^*n*^(*x*),*τ*^*n*^(*y*)] = 0 for all *v* ∈*U.* This yields that 2.22

Combining (2.20), (2.21) and (2.22) we find that *Φ*_*n*_(*u*,*v*)[*τ*^*n*^(*x*),*τ*^*n*^(*y*)] = 0 i.e., *τ*^−*n*^(*Φ*_*n*_(*u*,*v*))[*x*,*y*] = 0. Hence, we conclude that *Φ*_*n*_(*u*,*v*) = 0 for all *u*,*v*∈*U.*This completes the proof of our theorem. □

Some special cases of the above theorem are already known and are of great interest.

**Corollary 2.3.** ((Ashraf et al. 2004), Theorem 2.3) Let *R* be a 2 torsion free prime ring and *U* a square closed Lie ideal of *R*. Suppose that *σ*,*τ* are endomorphisms of *R* such that *τ* is one-one, onto. Suppose further that *U* has a commutator which is not a zero divisor. If *F*:*R*→*R* is a generalized Jordan (*σ*,*τ*) derivation of *U* into *R* then *F* is a generalized (*σ*,*τ*) derivation of *U* into *R*.

**Corollary 2.4.** ((Cortes and Haetinger 2005), Theorem 1.3) Let *R* be a 2 torsion free ring such that *R* has a commutator which is not a right divisor and *U* a square closed Lie ideal of *R*. Then every generalized Jordan higher derivation of *U* into *R* is a generalized higher derivation of *U* into *R*.
